# From Psychoactivity to Antimicrobial Agents: Multifaceted Applications of Synthetic Cathinones and *Catha edulis* Extracts

**DOI:** 10.3390/molecules29245918

**Published:** 2024-12-15

**Authors:** Celia María Curieses Andrés, José Manuel Pérez de la Lastra, Elena Bustamante Munguira, Celia Andrés Juan, Eduardo Pérez-Lebeña

**Affiliations:** 1 Hospital Clínico Universitario of Valladolid, Avenida de Ramón y Cajal, 3, 47003 Valladolid, Spain; cmcuriesesa@saludcastillayleon.es (C.M.C.A.); ebustamante@saludcastillayleon.es (E.B.M.); 2 Institute of Natural Products and Agrobiology, CSIC-Spanish Research Council, Avda. Astrofísico Fco. Sánchez, 3, 38206 La Laguna, Spain; 3 Cinquima Institute and Department of Organic Chemistry, Faculty of Sciences, Valladolid University, Paseo de Belén, 7, 47011 Valladolid, Spain; 4 Valladolid University Foundation, Valladolid University, Paseo de Belén, 11, 47011 Valladolid, Spain; info@glize.eu

**Keywords:** drug design, synthetic cathinones, poisoning, chirality enantioselectivity, enantioseparation

## Abstract

The emergence of new psychoactive substances (NPS) in the global drug market since the 2000s has posed major challenges for regulators and law enforcement agencies. Among these, synthetic cathinones have gained prominence due to their stimulant effects on the central nervous system, leading to widespread recreational use. These compounds, often marketed as alternatives to illicit stimulants such as amphetamines and cocaine, have been linked to numerous cases of intoxication, addiction and death. The structural diversity and enantiomeric forms of synthetic cathinones further complicate their detection and regulation and pose challenges to forensic toxicology. In addition to their psychoactive and toxicological effects, new research suggests that cathinones may have antimicrobial properties. Compounds derived from *Catha edulis* (khat), including cathinone, have shown antimicrobial activity against multidrug-resistant bacteria such as *Staphylococcus aureus* and *Escherichia coli*, highlighting their potential role in the fight against antibiotic resistance. This article provides an overview of the chemistry, pharmacokinetics, pharmacodynamics, toxicological effects and potential antimicrobial applications of synthetic cathinones. The potential therapeutic use of cathinone-derived compounds to combat antimicrobial resistance represents an exciting new frontier in drug development, although further research is needed to balance these benefits with the psychoactive risks.

## 1. Introduction

Cathinone is a naturally occurring stimulant found in the leaves of the khat plant (*Catha edulis*), which is native to the Horn of Africa and parts of the Arabian Peninsula. In these regions, khat consumption is deeply embedded in cultural and social traditions and is often consumed by chewing or as tea [1,2]. Over time, this practice has also spread to Europe and North America. The active constituents of fresh khat leaves include a variety of alkaloids, flavonoids, amino acids, glycosides, sterols, vitamins and minerals [3,4].

**Figure 1:**
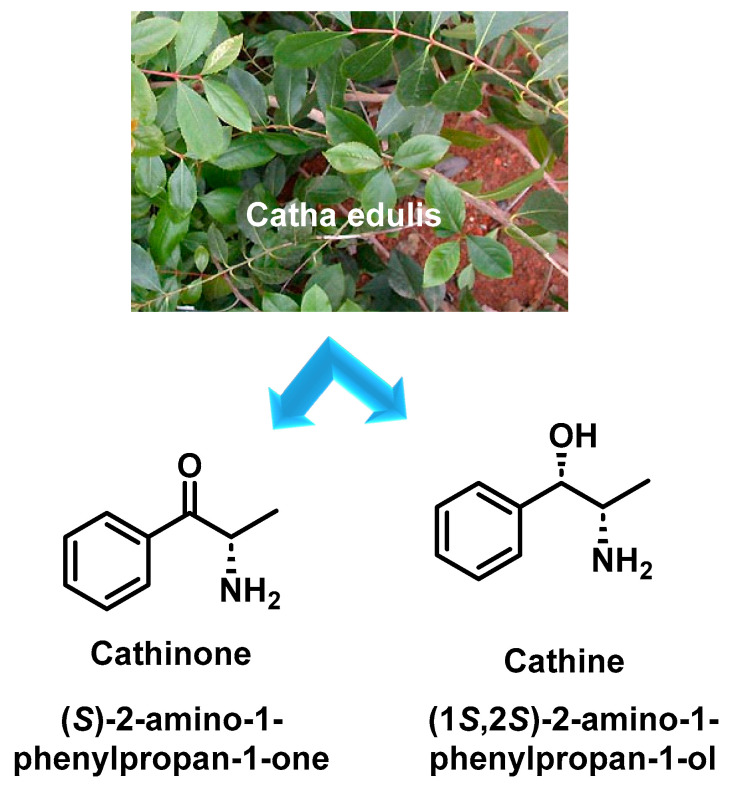
Plant of khat and chemical structures of cathinone and cathine.

In the 1930s, cathine (norpseudoephedrine) was identified as one of the active constituents of khat. Later, in 1975, cathinone was isolated and was found to be much more bioactive than cathine and to have similar psychoactive effects to amphetamines due to its structural similarity [5]. Cathinone can occur in two enantiomeric forms, with the (S)-(−)-enantiomer being primarily responsible for its pharmacological effects (Figure 1).

Over time, as the khat leaves age or dry out, cathinone degrades into less potent compounds such as cathine and (−)-norephedrine. To experience the full stimulant effect, khat leaves must therefore be consumed shortly after harvest.

**Figure 2:**
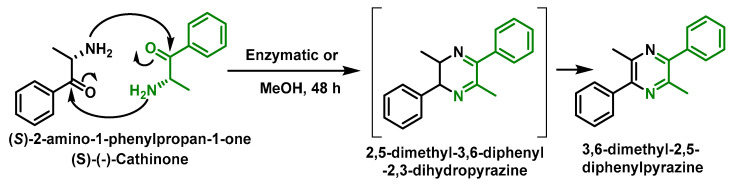
Formation of 3,6-dimethyl-2,5-diphenylpyrazine.

In different studies, cathinone has been found to be chemically unstable and very labile [6]. These chemical characteristics may explain why cathinone was not identified earlier. In the 2000s, cathinone appeared on Israeli recreational markets in 200 mg capsules, under the brand name ‘Hagigat’, which was sold as a natural psychostimulant and aphrodisiac [7].

(S)-(−)-cathinone can be dimerized to produce 3,6-dimethyl-2,5-diphenylpyrazine, making it difficult to purify and isolate [8] (Figure 2).

Cathinone tends to be cycled to 3,6-dimethyl-2,5-diphenyldihydropyrazine with subsequent oxidation to 3,6-dimethyl-2,5-diphenylpyrazine [9]. Dimerization has been reported to occur at room temperature and after base extraction and evaporation to dryness [9,10].

For this study, a detailed literature review was conducted in several databases, including PubMed, Scopus Springer Link, SciELO, Web of Science, and official websites of international organizations, using general terms such as, “bath salts”, “designer drugs”, “legal drugs”, “novel psychoactive substances”, “research chemicals”, “synthetic cathinones”, “cathinone derivatives”, “enantiomerically pure synthetic cathinones, as well as the specific names of the different cathinones most used in the last 10 years.

## 2. Synthetic Cathinones

**Figure 3:**
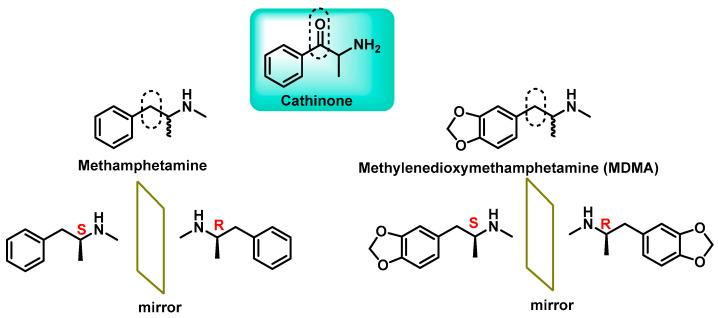
Chemical structure of cathinone, methamphetamine and methylenedioxymethamphetamine and the stereoisomers.

Synthetic cathinones first emerged as legal alternatives to substances such as amphetamines, ecstasy and cocaine. Originally developed for therapeutic purposes, these compounds have since been modified to circumvent legal controls, resulting in a wide range of derivatives available on the illicit market [11]. Structurally, synthetic cathinones are β-keto-phenethylamines, which are closely related to methamphetamine and methylenedioxymethamphetamine (MDMA). The primary structural difference lies in the presence of a ketone group at the β-position of the aminoalkyl chain [12]. For this reason, these “legal drugs” are often referred to as βk-amphetamines [13] (Figure 3).

### 2.1. Availability of Synthetic Cathinones

Synthetic cathinones are often sold under various brand names such as Blue Silk, White Knight and Vanilla Sky. These substances are marketed as “bath salts”, “plant food” or “herbal incense” or fertilizers to avoid legal controls [14,15,16]. They are usually available in the form of powders or capsules and often contain multiple active ingredients or adulterants such as caffeine or local anesthetics [17,18]. The constant change in their chemical structure means that new derivatives are constantly appearing, complicating efforts to regulate and detect these substances. The pharmacological mechanisms of action of synthetic cathinones are similar in nature to those exerted by traditional psychostimulants [19]. They are inhibitors of the presynaptic reuptake transporters of dopamine and norepinephrine [20].

### 2.2. Routes of Administration

Synthetic cathinones are typically consumed orally, either individually or mixed with other substances [21,22]. Common oral methods include swallowing capsules or tablets, ingesting the powder wrapped in cigarette paper (a method known as “bombing”), or mixing the powder into a drink. Nasal insufflation (snorting) is another popular method, often involving “keying”, where users dip a key into the powder and inhale it. Additionally, intravenous injection (also called “slamming”) has recently become a frequent route of administration for these drugs of abuse [23,24].

While less common, synthetic cathinones can also be administered through other methods [25], such as

- Intramuscular or subcutaneous injection,
- Rectal insertion (often called “booty bumping” or “plugging”),
- Gingival or sublingual absorption,
- Inhalation (smoking or vaping with e-cigarettes), and
- Ocular insertion (“eyeballing”).

It is also noted that users may combine multiple routes in a single session to enhance effects. Regardless of the consumption method, the psychoactive effects are generally similar. For instance, mephedrone users report that after nasal insufflation, effects begin within 10–20 min and last 1–2 h, while oral ingestion leads to effects within 15–45 min, lasting 2–4 h. Intravenous injection provides a rapid onset, with peak effects occurring within 10–15 min and lasting approximately 30 min [26,27].

Synthetic cathinones are often used in conjunction with other substances, including amphetamines, cocaine, cannabinoids, alcohol, or prescription medications, to intensify the psychoactive experience [28]. The amount consumed per session can range from a few milligrams to several grams, depending on factors such as the cathinone derivative, the method of administration, and the purity and concentration of the drug [29]. While oral administration is preferred over nasal insufflation, many users cite the latter’s tendency to irritate the nasal mucosa, especially with derivatives like mephedrone and 3,4-dimethylmethcathinone (3,4-DMMC) [30].

Users cite several reasons for using synthetic cathinones, for example their (i) legality (as they offer an alternative to controlled substances) [29,31], (ii) accessibility (they are readily available online). Affordability (lower cost relative to conventional drugs), (iii) evasion of detection (lack of rapid tests for synthetic cathinones makes them harder to detect) [32], (iv) enhanced social and sexual experiences (users believe these substances improve sociability and sexual encounters), (v) appealing presentation (attractive branding and packaging are enticing), and (vi) legal euphoria (they are often incorrectly regarded as both legal and safe to be consumed, with presumed high purity) [25].

### 2.3. Classification of Synthetic Cathinones

**Figure 4:**
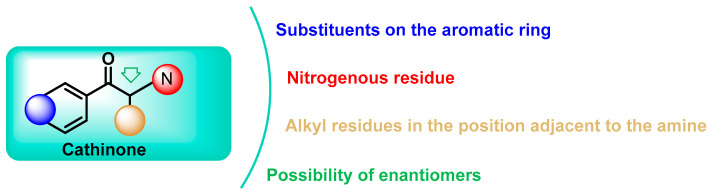
General structure of synthetic cathinones.

Chemical modifications and functional group substitutions of the cathinone structure have resulted in a large number of new synthetic cathinone psychostimulants, the number of structural modifications has been growing, making them the second largest group of new psychoactive substances in Europe [5,33]. The chemical structure of cathinones allows for numerous modifications, resulting in a wide variety of synthetic derivatives [34] (Figure 4).

Based on these structural modifications. These can be broadly classified into four main types based on their chemical structure: N-alkyl cathinones, N-pyrrolidine cathinones, 3,4-methylenedioxy-N-pyrrolidine cathinones, and 3,4-methylenedioxy-N-alkyl-cathinones [5,35]. Each of these categories comprises numerous substances with different pharmacological effects and degrees of toxicity [35].

#### 2.3.1. N-alkyl Cathinones

**Figure 5:**
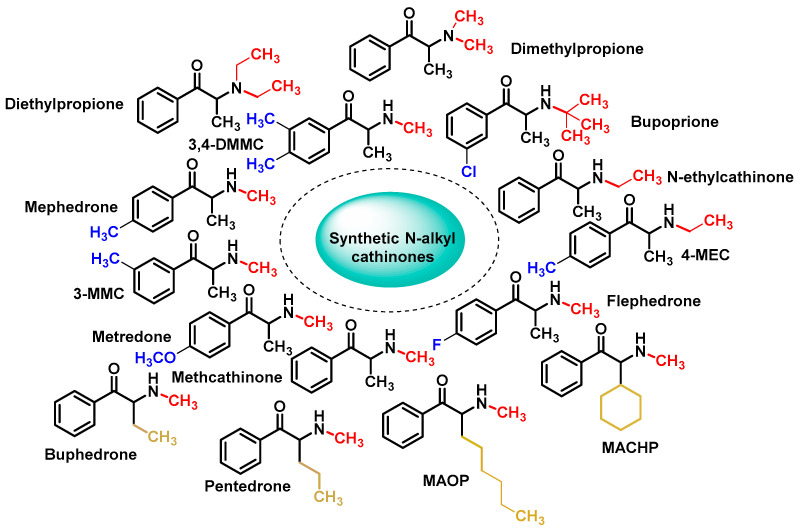
Chemical structure of synthetic N-alkylated cathinones.

The first generation of synthetic cathinones, the N-alkyl cathinones, comprises compounds with one or no substituents on the aromatic ring and/or alkyl substitutions on the α-carbon of the side chain [36] (Figure 5).

Methcathinone, one of the first cathinone derivatives synthesized for medical purposes, was originally developed as an antidepressant but later became notorious for its addictive properties [5,37]. It became a popular street drug in the 1970s in the Soviet Union and in the 1990s in the USA, where it was known by names such as “Cat” or “Jeff” [38,39].

Bupropion hydrochloride is the only synthetic cathinone derivative currently used as an antidepressant and support for smoking cessation [40,41]. Marketed under the name Wellbutrin. Bupropion has been shown to be an effective antidepressant that acts as a dopamine and norepinephrine reuptake inhibitor [42,43]. Chemically unrelated to typical antidepressants (tricyclic antidepressants and selective serotonin reuptake inhibitors), bupropion manages to avoid common adverse side effects such as sexual dysfunction, weight gain, and drowsiness [44,45]. Sold under the name Zyban. In 2019, bupropion was ranked 22nd among the most prescribed drugs in the U.S. and has since been added to the World Health Organization’s Model List of Essential Medicines [13,46].

Bupropion is effective in preventing depressive episodes in patients with seasonal affective disorders and can be used for the treatment of attention deficit hyperactivity disorder and psychoactive substance dependence [47].

**Figure 6:**
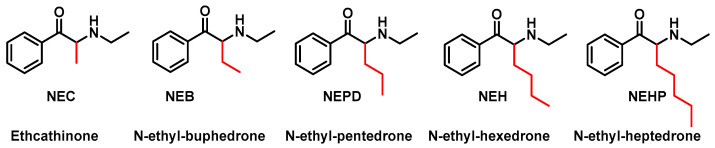
Chemical structure of NEC, NEB, NEPD, NEH and NEHP.

In 2023, Nadal-Gratacós et al. synthesized and studied the in vitro and in vivo effects of five N-ethyl-substituted cathinones that differ only in the length of the alpha-carbon side chain. They are N-ethyl-cathinone (NEC), N-ethyl-bufedrone (NEB), N-ethyl-pentedrone, N-ethyl-hexedrone (NEH) and N-ethyl-heptedrone and they are able to inhibit dopamine uptake and are selective for dopamine transport [48] (Figure 6).

The potency of DA uptake inhibitors increases with methyl-to-propyl aliphatic side chain size and decreases when butyl to pentyl is increased, which correlates with an inverted U-shaped psychostimulant response in mice at the average dose tested. On the other hand, an increase in the length of the α carbon side chain correlates with an increase in cytotoxic properties in PC12 cells, probably due to better membrane penetration [39].

These synthetic cathinones have recently been identified in the illicit drug market, causing poisoning and even death with their use and abuse [49]. N-ethyl-hexenone has been classified as a Schedule II controlled substance under the United Nations Convention on Psychotropic Substances [50].

#### 2.3.2. N-pyrrolidine Cathinones

**Figure 7:**
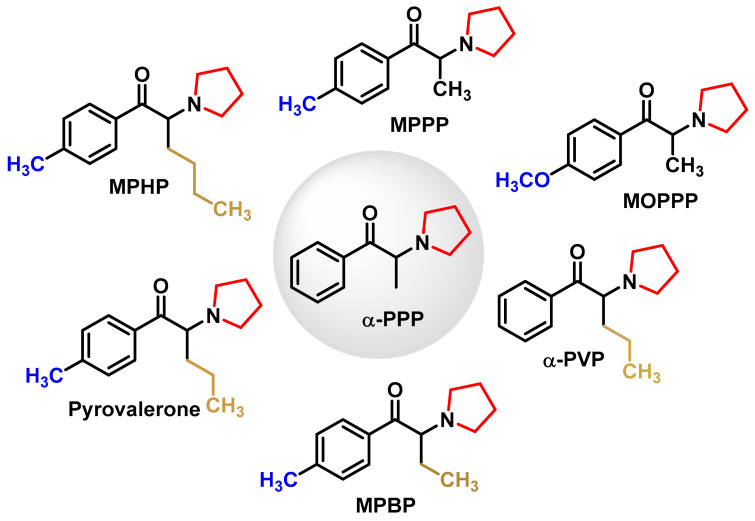
Chemical structures of N-pyrrolidine cathinone derivatives. α-PPP (α-pyrrolidinopropiophenone).

N-pyrrolidine cathinones have a pyrrolidine ring, a side chain with different numbers of carbon atoms at the alpha position and monosubstitution in the aromatic ring [51] (Figure 7).

N-pyrrolidine cathinones gained notoriety due to their potent psychoactive effects and addictive properties [52]. Cathinone N—replaced by pyrrolidine derivatives of α-PPP (α-pyrrolidininolphenone)—appeared on the German drug market. Pyrovalerone derivatives, first synthesized in the 1970s to treat conditions such as chronic fatigue and obesity, were eventually withdrawn from clinical use after reports of abuse and addiction [53].

One particularly notorious example is α-PVP, known on the street as “flakka” or “gravel”. This drug, notorious for its severe stimulant effects, including excitatory delirium and psychosis, became an epidemic in South Florida [54]. Its potent inhibition of dopamine reuptake mimics cocaine and leads to intense euphoria, but also carries significant psychiatric risks. Unlike typical drug tests, α-PVP is difficult to detect and requires advanced methods such as gas chromatography and mass spectrometry [55].

Derivatives such as α-PVT and naphyrone (which is sold as “NRG-1”) have also been shown to be potent stimulants, providing further evidence of how small structural changes in cathinone derivatives can create new psychoactive substance classes with different effects on the body’s dopamine system [41].

**Figure 8:**
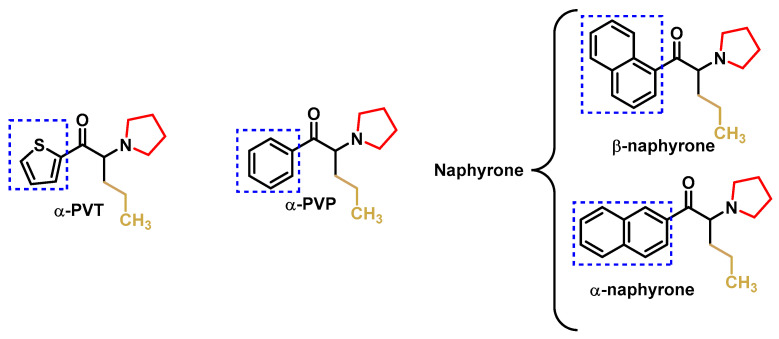
Structural formulae of α-PVT and naphyrone.

The replacement of the phenyl ring in α-PVP with other aromatic rings, such as thiophene, provided alpha-pyrrolidinopenthiothiothiophenone (α-PVT) or the naphthalene known as naphyrone (naphthylpyrovalerone, O-2482), which is a mixture of alpha and beta isomers and not just the β isomer, as described in the literature [56] (Figure 8).

Naphyrone (naphthylpyrovalerone was marketed in the UK under the name “Energy-1” (NRG-1) as a legal alternative to mephedrone [41,57].

#### 2.3.3. 3,4-Methylenedioxy-N-pyrrolidine Cathinones

**Figure 9:**
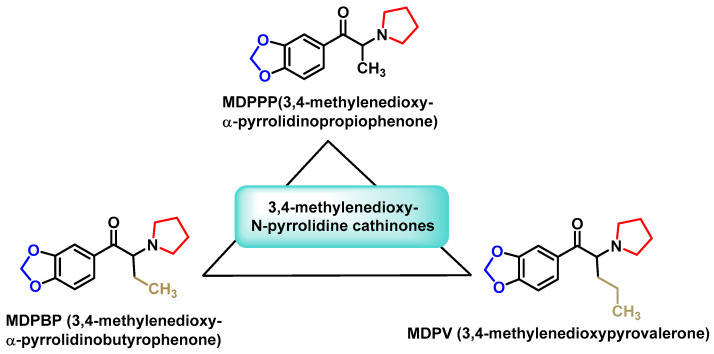
Chemical structure of 3,4-methylenedioxy-N-pyrrolidine derivatives.

This group combines the structural features of a 3,4-methylenedioxy ring with an N-pyrrolidinyl unit (Figure 9). One of the best-known substances in this category is 3,4-MDPV, a compound first synthesized in 1969 for the treatment of chronic fatigue. Despite its medicinal origin, 3,4-MDPV quickly became notorious for its high potential for abuse and dependence [58].

The lipophilicity of the substance allows it to cross the blood–brain barrier efficiently, meaning that only small doses are required to produce a strong psychoactive effect. Known as “Magic”, “Super Coke”, “Peevee”, “New Ivory Wave” and “Bath salts”, it was first detected in 2007 in Japan [59] {Uchiyama, 2008 #12412}. For years, it was the most popular of all pyrovalerone derivatives in Europe, the US and Japan, and even the most widely abused synthetic cathinone in some EU countries [60,61,62].

#### 2.3.4. 3,4-Methylenedioxy-N-alkyl Cathinones

**Figure 10:**
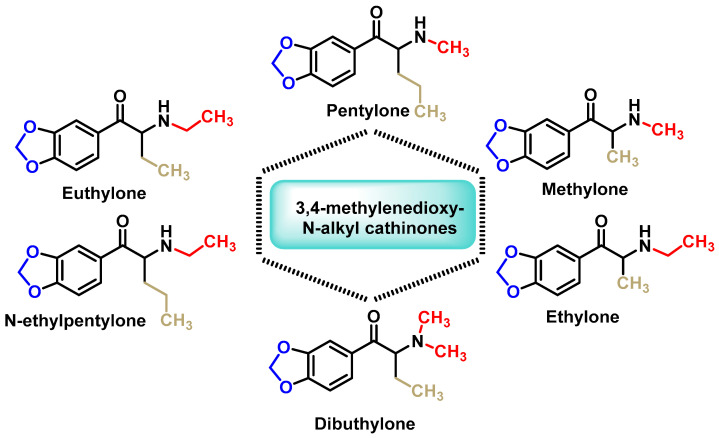
Chemical structure of 3,4-methylenedioxy-N-alkyl derivatives.

These derivatives are characterized by the presence of a 3,4-methylenedioxy group attached to the aromatic ring and alkyl substitutions on the nitrogen atom (Figure 10). Methylone, one of the first synthetic cathinones in this category, was initially synthesized in 1996 as an antidepressant and anti-Parkinson’s drug, but was never marketed for medical use [63].

Instead, methylone emerged on the recreational markets in the early 2000s under names such as “explosion”, where it became popular for its MDMA-like effects. Methylone inhibits the reuptake of dopa-mine, noradrenaline and serotonin, albeit with less potency than MDMA itself. Nevertheless, cases of intoxication with methylone, both alone and in combination with other psychoactive substances, are widespread [64].

#### 2.3.5. Stereoisomers of Synthetic Cathinones

**Figure 11:**
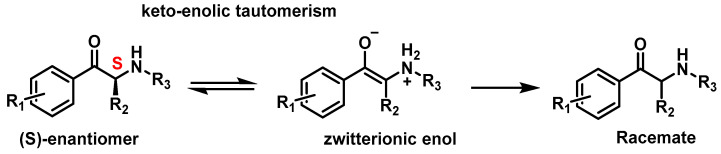
Tautomerism of synthetic cathinones.

Cathinones possess a stereocenter, making them chiral molecules, which means they exist as two enantiomers (R and S forms). These enantiomers can exhibit different potencies and affinities toward their pharmacological targets. In biological systems, such as the human body, enantiomers often display distinct activities and vary in the intensity of their effects [65]. Typically, one enantiomer may be more pharmacologically active, while the other may contribute to adverse effects or even higher toxicity. Despite this, most synthetic cathinones (SCs) are distributed as racemic mixtures, containing equal amounts of both enantiomers. Additionally, racemization between enantiomeric forms can occur through keto–enol tautomerism [35,65] (Figure 11).

The differentiation between the (R) and (S) enantiomers is critical in both clinical and forensic toxicology. For instance, in naturally occurring cathinone, the S-enantiomer is the primary active compound found in khat leaves and is responsible for its potent central nervous system (CNS) stimulation [66]. This enantiomeric trend has been confirmed in several synthetic cathinones like methcathinone, α-PVP, and MDPV. Studies on animal models, particularly rats, have demonstrated that the S-enantiomer typically exhibits stronger stimulatory effects. Many reports attribute the primary stimulatory properties of cathinones to the S-(−) enantiomer.

For example, research by Glennon et al. revealed that the S-enantiomer of methcathinone displayed stronger CNS stimulatory effects compared to the R-enantiomer [67]. Davies et al. further explored the effects of methcathinone enantiomers on monoamine transporters [68], while Gregg et al. examined the neurochemical effects of mephedrone enantiomers in rats [69]. Hutsell et al. investigated the distinct responses of 4-methylcathinone enantiomers on monoamine release and behavioral effects using intracranial self-stimulation (ICSS) in rats [70]. Similarly, Kolanos et al. showed that the S-(+)-enantiomer of MDPV was more potent in inhibiting dopamine and norepinephrine reuptake and facilitated intracranial self-stimulation [71].

Further studies by Schindler et al. focused on the neurochemical, behavioral, and cardiovascular effects of α-PVP enantiomers in rats, corroborating the higher potency of the S-enantiomer [72]. In terms of drug absorption and metabolism, Silva et al. found that the R-(−) pentedrone enantiomer and the S-(−) methylone enantiomer exhibited greater permeability through the gastrointestinal tract in a Caco-2 cell model. However, subsequent findings from the same group indicated that the S-(+) pentedrone enantiomer and R-(+) methylone enantiomer were the most oxidative and cytotoxic forms [73].

These studies are essential for identifying which enantiomers are responsible for the major biological or toxicological effects of cathinones, particularly in cases of abuse. Understanding the differential activity of cathinone enantiomers could play a crucial role in evaluating their potency, toxicity, and potential for dependence [74].

## 3. Chemical Synthesis of Cathinones

Synthetic cathinones are typically produced in racemic mixtures, where both enantiomers are present in equal proportions [75]. Their synthesis in the laboratory is straightforward and generally involves the following methods:

(a) Starting from an aryl ketone,
(b) Neber rearrangement, and
(c) Modified Neber rearrangement.

Each method yields cathinones with high purity.

### 3.1. Synthesis from Aryl Ketones

The synthesis process for cathinones via aryl ketones involves three key steps: selection of a suitable β-ketoarylalkane (aryl ketone), bromination at the alpha position relative to the keto group under acidic conditions, and amination using the desired amine [76].

**Figure 12:**
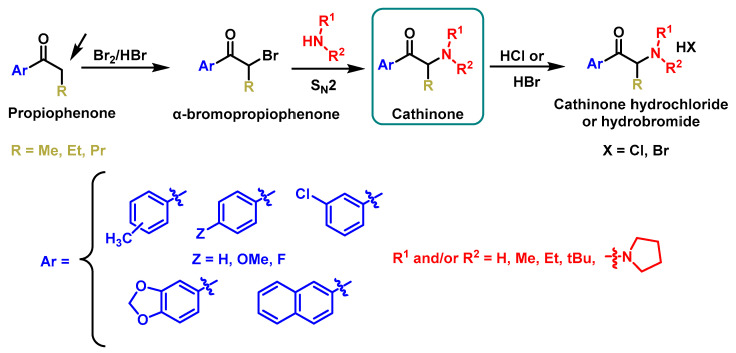
General method of synthesis of synthetic cathinones from aryl ketones.

This approach reliably produces cathinone derivatives with high purity as racemic mixtures. Due to the instability of the free base form, cathinones are typically isolated as their corresponding hydrochloride or hydrobromide salts [77] (Figure 12).

### 3.2. The Neber Rearrangement and the Modified Neber Rearrangement

An alternative synthesis method is the Neber rearrangement, which starts with an oxime derived from 1-phenyl-1-propanone. This compound undergoes reaction with sodium hydroxide to form a 2H-azirine intermediate, which is subsequently treated with aqueous acid to yield the corresponding α-aminoketone [78,79].

**Figure 13:**
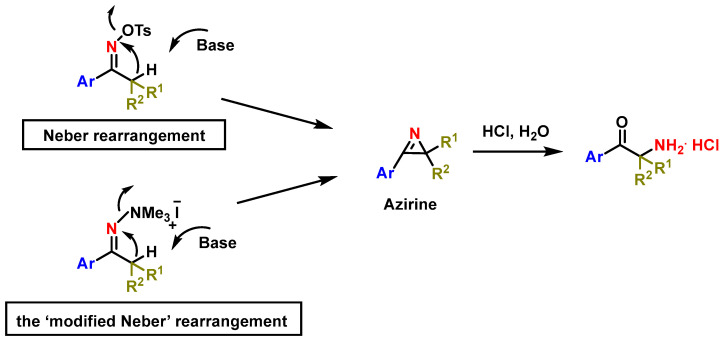
Generalized steps for the Neber rearrangement.

In the modified Neber rearrangement, the process involves quaternary hydrazones rather than oximes [80]. The final step, as with the standard Neber rearrangement, involves treatment with acid to form the α-aminoketone, which is then isolated as a hydrochloride salt [81,82,83,84,85,86] (Figure 13).

**Figure 14:**
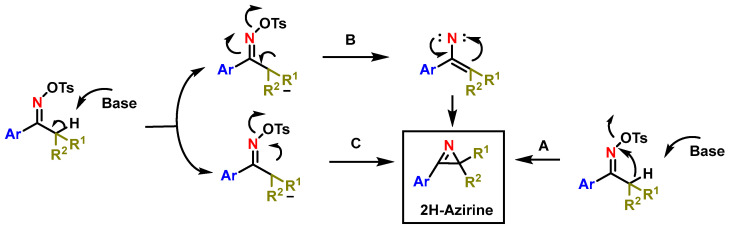
Possible mechanisms of formation of 2H-azirines by Neber rearrangement.

The mechanism for 2H-azirine formation is not fully understood, with three proposed pathways involving either a concerted process (A pathway) or an intermediate aza-allyl anion leading to 2H-azirine via a vinyl-nitrene (B pathway) or a carbon–nitrogen bond (C pathway) (Figure 14).

### 3.3. Synthesis of Enantiomerically Pure Cathinones

To synthesize enantiomerically pure cathinones, different strategies must be employed compared to racemic mixtures. Two primary approaches are:

- Enantioselective synthesis, which directly produces the desired enantiomer, and
- Enantiomeric resolution, which separates the enantiomers either directly or indirectly [38,87,88].

In the direct method, no prior derivatization is required. Conversely, in the indirect method, derivatization with enantiomerically pure reagents converts enantiomers into diastereoisomers, which can then be separated using crystallization or chromatography [89,90].

**Figure 15:**
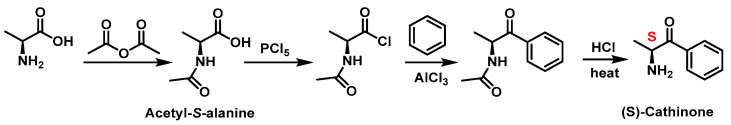
Synthesis of enantiomerically pure (S)-cathinone starting from (S)-alanine.

For example, S-(+)-cathinone can be synthesized from S-alanine via N-acetylation, followed by acyl chloride formation with phosphorus pentachloride. Friedel–Crafts acylation is then performed using aluminum chloride as a catalyst. The final step involves deacetylation using hydrochloric acid and heat (Figure 15).

**Figure 16:**
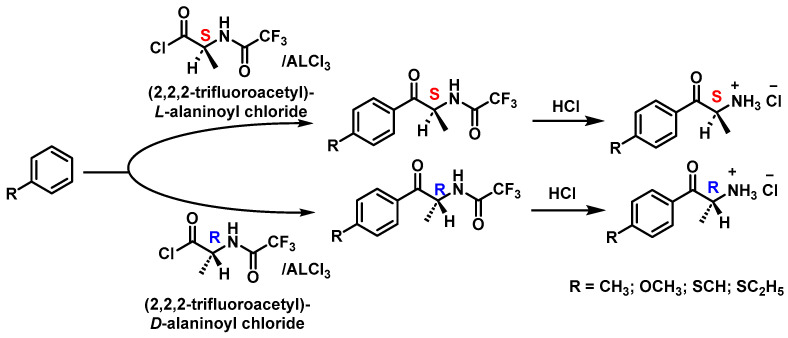
Synthesis of enantiomers of 4-methylcathinone, 4-methoxycathinone, 4-methylthiocathinone and 4-ethylthiocathinone (as hydrochloride salts).

This process has also been used to produce substituted cathinones, such as 4-methylcathinone, by similar acylation and hydrolysis steps [91] (Figure 16).

**Figure 17:**
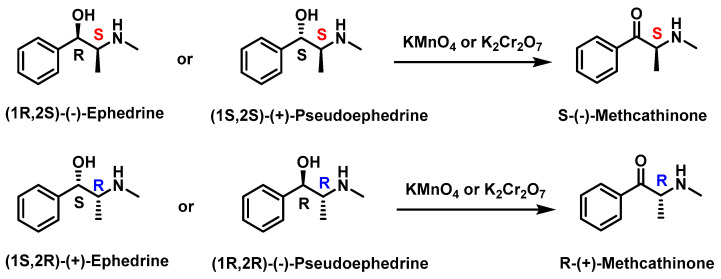
Synthesis of (S) and (R)-methcathinone through oxidation of ephedrines and pseudoephedrines.

Additionally, oxidation of ephedrine and pseudoephedrine with potassium permanganate or dichromate produces S-(−)-methcathinone or R-(+)-methcathinone, depending on the starting isomer [92] (Figure 17).

An alternative method for synthesizing optically active cathinone involves creating diastereomers by derivatizing a racemic mixture with an enantiomerically pure reagent. This process forms a covalent bond, resulting in diastereomers that can then be separated under achiral conditions using techniques like crystallization or chromatography.

**Figure 18:**
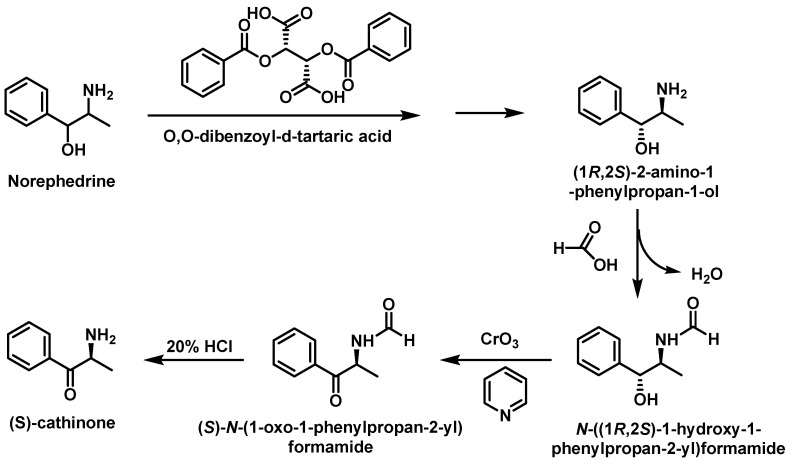
Synthesis of (S)-(−)-cathinone by resolution of norephedrine.

To obtain (S)-(−)-cathinone, norephedrine is resolved with O,O-dibenzoyl-tartaric acid, converting the (S)-(−) enantiomer into its N-formyl derivative. This is then oxidized with chromium trioxide in pyridine. The final step, hydrolysis with hydrochloric acid, yields (S)-(−)-cathinone with a moderate yield of approximately 39% [9] (Figure 18).

### 3.4. Analytical Techniques for Enantiomeric Separation

Chiral separation of synthetic cathinones can be achieved using two main techniques:

- Direct separation on chiral stationary phases (CSPs) or using chiral additives in the mobile phase [93,94], and
- Indirect separation via derivatization into diastereomers, which are separated under achiral conditions [95,96,97,98,99,100].

**Figure 19:**
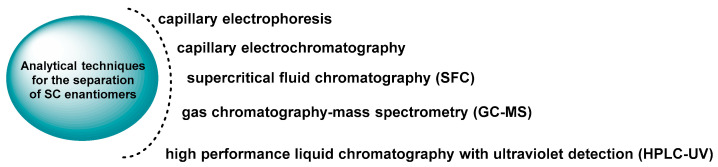
Analytical techniques for the separation of SC enantiomers.

Chiral derivatization is more cost-effective and provides good resolution for diastereomers [101], whereas CSPs offer advantages such as simplicity [102], time efficiency, and better enantioseparation for compounds like tertiary amines that are challenging to separate by derivatization [89,103,104] (Figure 19).

#### 3.4.1. Capillary Electrophoresis (CE)

**Figure 20:**
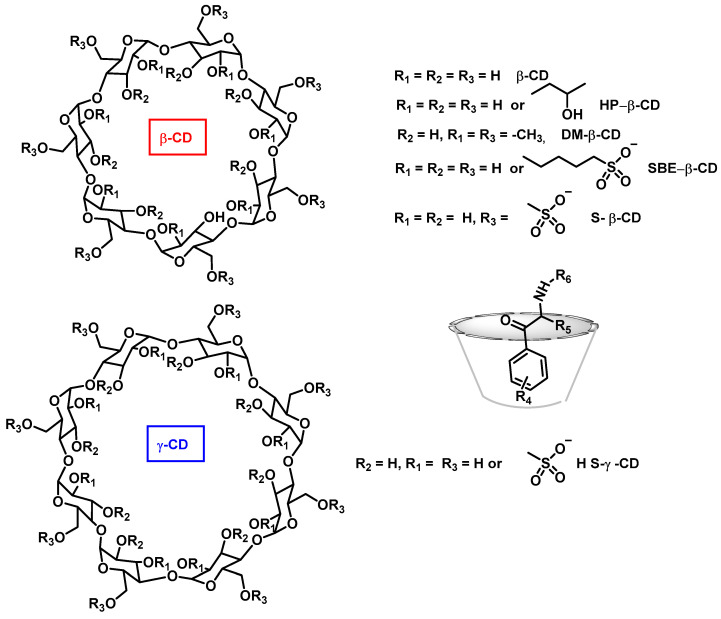
Cyclodextrins used in capillary electrophoresis for the separation of synthetic cathinones.

Capillary electrophoresis (CE) offers a complementary separation technique to HPLC, where analytes are separated by their migration rates under an electric field. CE is widely used for chiral analysis of cathinones, with cyclodextrins (CDs) often acting as chiral selectors [105,106,107]. Studies have shown that 2-hydroxyethyl-β-CD offers the best enantioselectivity for certain cathinones such as methcathinone, mephedrone, and 3-MMC [105,106,108,109] (Figure 20).

#### 3.4.2. Gas Chromatography (GC)

**Figure 21:**
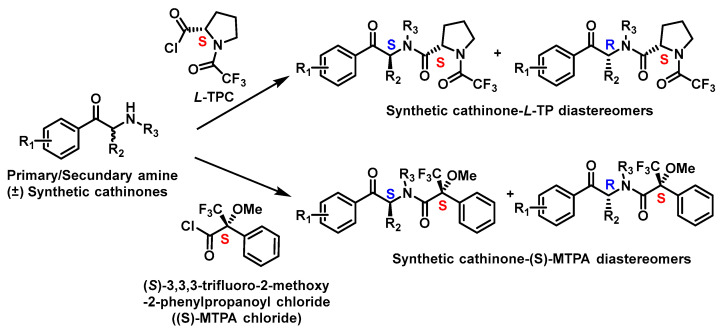
Chiral derivation of synthetic cathinones from primary or secondary amines by L-TPC and (S)-MTPA chloride.

For gas chromatography (GC), an indirect approach is most common due to limited availability of suitable chiral stationary phases (CSPs). Derivatization agents, such as (S)-MTPA chloride or L-TPC, are typically used to transform the cathinones into diastereomers for improved resolution [110] (Figure 21).

#### 3.4.3. Liquid Chromatography Using CSPs

High-performance liquid chromatography (HPLC) using chiral stationary phases (CSPs) is a widely employed technique for separating synthetic cathinone enantiomers due to the variety of commercial CSPs available [38,96]. These CSPs are based on various chiral selectors, including crown ethers, cyclodextrins, proteins, antibiotics, and polysaccharides [94,111,112,113,114,115]. This method is favored because it can be coupled with multiple detection modes, such as ultraviolet–visible (UV–vis) absorption and mass spectrometry (MS).

In recent studies, Paskan et al. (2024) achieved chiral separation of synthetic cathinone enantiomers derived from α-tetralone using HPLC equipped with a circular dichroism (CD) detector, which enabled them to obtain CD spectra of the separated enantiomers [116]. HPLC with CSP is therefore considered a practical and versatile option for both analytical and preparatory purposes [117]. Recently, ultra-high-performance liquid chromatography (UHPLC) has gained attention for its improved selectivity, higher efficiency, and shorter analysis time compared to HPLC, and CSPs that are adaptable to UHPLC are now available [118,119].

**Figure 22:**
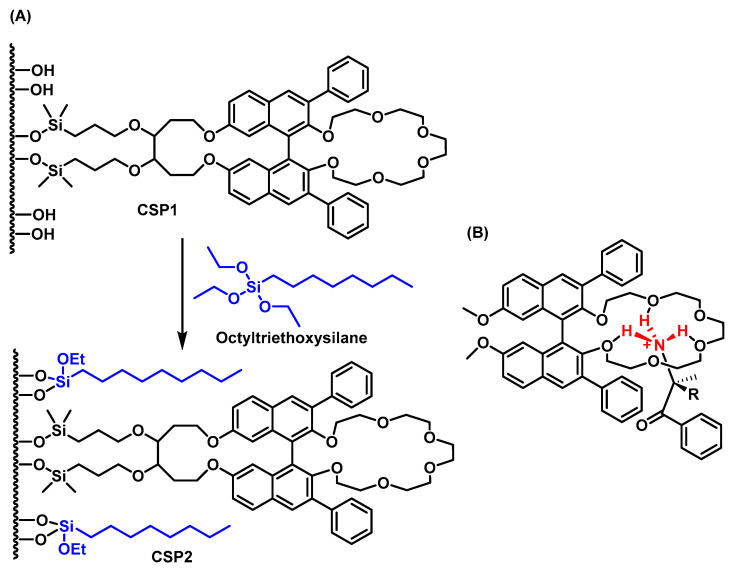
(**A**) Crown ether phase, (3,3′-diphenyl-1,1′-bonapthyl)-20-crown-6 and reaction with n –octyltriethoxysilane and (**B**) possible mode of chiral recognition of cathinones.

Crown ether-based CSPs provide excellent resolution for cathinone enantiomers, particularly those derived from primary amines. However, CSP1 (unmodified crown ether-based CSP) contains unreacted silanol groups on the silica surface that can result in non-specific hydrogen bonding, reducing enantioselective chiral recognition. To address this, the residual silanol groups can be masked by treatment with n-octyltriethoxysilane in refluxing toluene, producing CSP2 (Figure 22), which both protects the silanol groups and improves the CSP’s lipophilicity, enhancing its capacity for chiral recognition [120].

**Figure 23:**
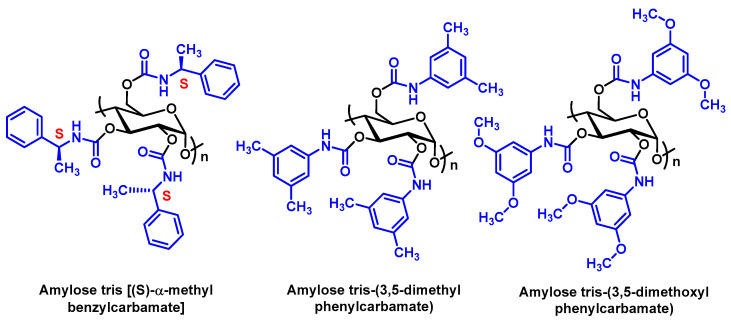
Chemical structures of polysaccharide-based chiral selectors.

Polysaccharide-based CSPs, like tris[(S)-α-methylbenzylcarbamate] amylose (e.g., Chiralpak AS-H) [94,121], have shown good results for enantiomer separation of synthetic cathinones with primary and secondary amines, although they are less effective for tertiary amine enantiomers. For cathinones containing a trifluoromethyl group on the aromatic ring, separation of enantiomers can be achieved with ChiralART Amylose-SA polysaccharide-based CSP [122]. 3,4-Methylenedioxypyrovalerone (MDPV), a pyrrolidine-based cathinone, has also been successfully separated using polysaccharide CSPs, such as tris-3,5-dimethylphenylcarbamate amylose and tris-3,5-dimethoxyphenylcarbamate amylose [107] (Figure 23).

Meetani et al. quantified enantiomers of 18 synthetic cathinones with a tertiary amine structure using HPLC–UV–vis with Astec Cellulose DMP and Chiralpak AS-H amylose columns. The mobile phase consisted of hexane, isopropanol, and triethylamine [123].

**Figure 24:**
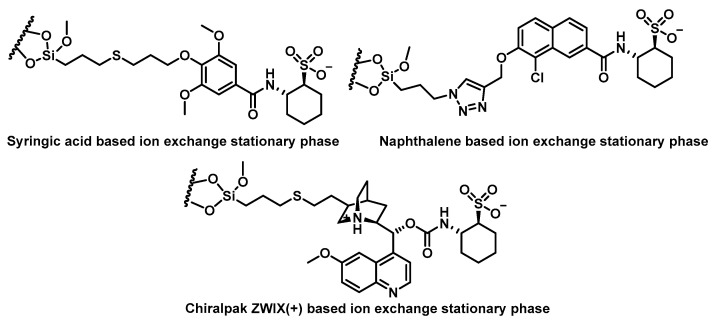
Three types of ion exchange CSPs for chiral separation.

Chiral ion exchange CSPs have also shown strong results in synthetic cathinone enantioseparation by HPLC. Wolrab et al., 2016, successfully separated 14 cathinone derivatives using three types of ion exchange CSPs [93]:

- A commercially available chiral zwitterionic ion exchanger, Chiralpak ZWIX(+),
- A chiral strong cation exchanger based on syringic acid [124], and
- A novel naphthalene-based strong cation exchanger (c-SCX) (Figure 24).

#### 3.4.4. Supercritical Fluid Chromatography (SFC)

Supercritical fluid chromatography (SFC) is an effective technique for the rapid separation of enantiomers, often providing faster results than high-performance liquid chromatography (HPLC). Many chiral stationary phases (CSPs) initially developed for HPLC are also compatible with SFC without additional modifications. However, SFC involves higher costs and requires more complex equipment, which may limit its accessibility.

Using the commercial ChiralArt Amylose SA column containing amylose tris(3,5-dimethylphenylcarbamate) as a chiral selector, rapid enantioseparation of synthetic cathinones such as mephedrone, brephedrone, and phlephedrone has been successfully demonstrated [125]. In 2015, Pauk et al. applied ultra-high-performance supercritical fluid chromatography for the separation of polar synthetic cathinones, utilizing a mobile phase composed of carbon dioxide, nitrous oxide, and various additives [126]. This study highlighted SFC’s efficiency in handling polar compounds, expanding its applicability in cathinone analysis.

#### 3.4.5. Capillary Electrochromatography (CEC)

Capillary electrochromatography (CEC) is a hybrid technique that combines elements of capillary electrophoresis (CE) and HPLC, with the mobile phase driven by electroosmosis, similar to CE. CEC separates analytes through partitioning between the mobile liquid phase and the stationary phase, as in HPLC [127,128].

Aturki et al. developed a chiral CEC method that achieved rapid enantiomeric separation of ten different cathinone derivatives within a few minutes. This method employed a chiral stationary phase housed in capillary columns with a 100 μm internal diameter, coated with silica-bound amylose tris(5-chloro-2-methylphenylcarbamate), also known as Sepapak 3 or Lux Amylose-2. This approach demonstrated CEC’s capability for high-speed, effective enantiomeric separation, making it a promising tool for synthetic cathinone analysis [129].

## 4. Pharmacological Classification of Synthetic Cathinones

Synthetic cathinones are generally classified as stimulants or amphetamine-type stimulants due to their action on the central nervous system [19]. The pharmacological profiles of these cathinone derivatives cover a wide spectrum, from those mimicking the effects of MDMA and cocaine, to others that exhibit methamphetamine-like psychostimulant properties, and highly dopaminergic derivatives such as the pyrovalerone cathinones [130].

Based on these varied effects, cathinones can also be classified according to their interaction with monoamine transporters specifically [5,38,130], norepinephrine transporters (NAT), serotonin transporters (SERT), and dopamine transporters (DAT) [63]. This classification is distinct from the structural classification based on chemical substitution patterns. The interaction with these transporters follows two primary mechanisms:

Inhibition of monoamine reuptake from the synaptic cleft, which occurs via binding to NAT, SERT, and/or DAT, prevents monoamines from being reabsorbed into presynaptic neurons.

**Figure 25:**
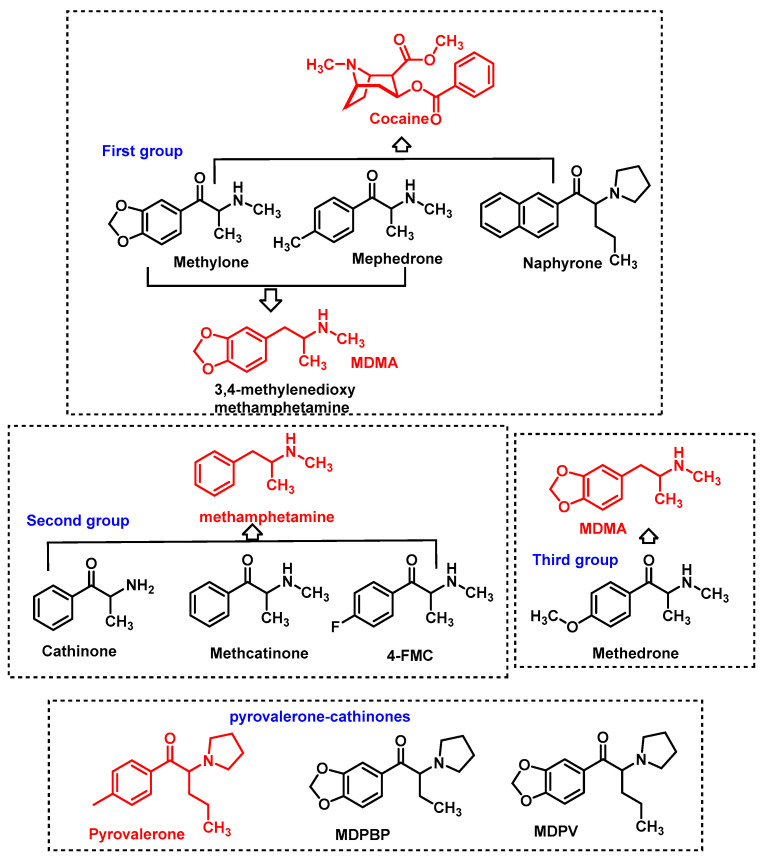
Classification of synthetic cathinones by pharmacological action.

Promotion of monoamine release increases the availability of these neurotransmitters in the synaptic cleft. Although these mechanisms are functionally opposite, both lead to an increase in extracellular monoamine levels, enhancing the signaling between neurons. The strength of interaction with each transporter can vary significantly between cathinone derivatives [13,131]. Consequently, a pharmacological classification system based on their mechanism of action has been proposed (Figure 25).

A classification of synthetic cathinones according to their pharmacological action can be divided into four groups:

- Non-selective Monoamine Uptake Inhibitors: Examples include methylone, mephedrone, and naphirone, which inhibit the reuptake of monoamines but exhibit a stronger affinity for DAT than for SERT, similarly to how cocaine operates. With the exception of naphirone, these cathinones also promote serotonin release, resembling the action of MDMA [40,132,133].
- Methamphetamine-like Cathinones: This group includes cathinone, methcathinone, and 4-fluoromethcathinone (4-FMC), which predominantly inhibit the reuptake of catecholamines (dopamine and norepinephrine) and act as dopamine-releasing agents. Their pharmacological profile closely aligns with methamphetamine.
- Serotonin-Norepinephrine Inhibitors with Low Dopamine Affinity: Cathinones in this category, such as methedrone and MDMA, display strong inhibition of NAT and SERT but have a relatively weak affinity for DAT.
- Pyrovalerone Derivatives: These cathinones, including α-PVP, are potent inhibitors of DAT and NET, but show weak or negligible activity at SERT. They are not substrates for monoamine transporters and have minimal affinity for monoamine receptors, making them highly dopaminergic without significant serotonergic effects.

### 4.1. Interaction of Monoamine Receptors and Transporters with Cathinones and Comparison with Related Amphetamines

Cathinones can interact with the transporters norepinephrine (NE), dopamine (DA), and serotonin (5-hydroxytryptamine [5-HT]) (NET, DAT, and SERT, respectively) to inhibit monoamine transport or induce transporter-mediated monoamine release [61]. Rickli et al., conducted a study to determine the effects of a series of para-(4)-substituted amphetamines and a series of pyrovalerone cathinones on monoamine uptake and release and interactions with various monoamine receptors [61]. A year earlier, Simmler et al. conducted the same study with MDMA, amphetamine, methamphetamine, methcathinone, mephedrone, phlephedrone, MDPV, naphironone, and pyrovalerone [19]. In 2014, Simmler et al., expanded the study of the in vitro pharmacology of a new series of cathinones, including methedron, 4-MEC, 3-FMC, pentilone, etcatinone, bufedrone, pentedron, and N,N-dimethylcathinone, and the profiles of non β-keto amphetamine analog comparator drugs 4-MTA, PMA, PMMA, MDMA, N-ethylamphetamine, and methamphetamine [130].

These researchers studied whether these compounds inhibit human NET, DAT, and SERT and determined the release mediated by NE, DA, and 5-HT transporters and characterized the binding affinities of the compounds for monoamine transporters, adrenergic receptors α 1 and α 2, dopamine receptors D1–D3, the serotonin receptors 5-HT 1A, 5-HT 2A and 5-HT 2C, the histamine receptor H 1 and the trace amine receptor associated 1 (TAAR 1).

For these studies, MDMA, cocaine or methamphetamine are taken as reference compounds. These reference compounds are ideal for the knowledge of their molecular mechanisms of action and for their pharmacological profiles with respect to their actions on monoamine systems.

### 4.2. Monoamine Transporter Inhibition

The potencies of drugs to inhibit SERT, NET, and DAT were evaluated in HEK 293 cells that stably expressed human SERT, NET, and DAT [134], as previously described [63,135]. The DAT/SERT ratio was calculated as 1/CI 50 of DAT:1/CI 50 of SERT.

Studying the NET, DAT and SERT values in Table 1, it is observed that MDMA is a potent NET and SERT inhibitor and with lower potency in DAT. Among the synthetic cathinones that resemble MDMA in their selectivity for SERT, there are methedrone, 4-ethylmethcathinone, 2,3-dimethylmethcathinone, 3,4-dimethylmethcathinone, and 2,4-dimethylmethcathinone. These cathinones inhibit high selectivity on SERT with respect to DAT inhibition. 4-Bromomethcathinone and 4-methylmethcathinone are also potent SERT inhibitors comparable to MDMA and are also quite potent DAT inhibitors. Pyrovalerone cathinones were very potent catecholamine transporter inhibitors (NET and DAT) with very low serotonergic activity, reflected by very high DAT: SERT inhibition ratios. Substitution of the 3,4-methylene ring found in MDMA and MDPV increased serotonergic activity compared to the unsubstituted compounds methamphetamine and α-PVP, respectively. Similarly, para-methylation in pyrovalerone increased the serotonergic property of the compound compared to α-PVP. However, in the case of pyrovalerones (MDPV and pyrovalerone), the inhibition potency of SERT was very low, even in the presence of these substitutions. The very high DAT: SERT inhibition ratio induced by pyrovalerone cathinones predicts particularly pronounced stimulant and addictive properties for this class of substances. In fact, MDPV and α-PVP are considered highly addictive.

As can be seen in Table 1, all compounds shared potent effects as NET inhibitors, and their DAT and SERT inhibition potencies varied considerably, which is reflected in the wide variation in DAT/SERT inhibition ratios. N,N-dimethylcathinone was a weak inhibitor of NET and DAT, Table 2.

Methamphetamine and MDMA were used as comparator compounds known to induce monoamine release. The specific binding of the radioligand to the target receptor was defined as the difference between total binding and non-specific binding determined in the presence of selected excess competitors. The following radioligands and competitors were used, respectively [3 H] 8-hydroxy-2-(di-n-propylamino) tetralin (8-OH-DPAT) and indatralin (5-HT1A receptor), [3 H] ketanserin and spiperone (5-HT 2A receptor), [3 H] ketanserin and spiperone (5-HT 2A receptor), [3H] mesulergine and mianserin (5-HT 2C receptor), [3H] prazosin and risperidone (α1-adrenergic receptor), [3H] rauwolscine and phentolamine (α2-adrenergic receptor).

Of the synthetic cathinones that act with high SERT potency, 5-HT can be expected to have subjective effects and intoxication profiles similar to those of MDMA or cocaine.

With the exception of 3,4-dimethylmethcathinone, all of these serotonergic cathinones also act as 5-HT releasers. 4-ethylmethcathinone, 4-bromomethcathinone, 4-methylmethcathinone, 2,3-dimethylmethcathinone, 3,4-dimethylmethcathinone and 2,4-dimethylmethcathinone showed binding to 5-HT2A receptors with similar or higher affinity than MDMA. In activity assays, 2,3-dimethylmethcathinone and 4-methylmethcathinone stood out as good 5-HT2A receptor activators, suggesting that they may cause hallucinations.

Several of these synthetic cathinones also show weak affinity at α1A- and α2A-adrenergic receptors.

## 5. Metabolism of Synthetic Cathinones

Synthetic cathinones are generally less efficient at crossing the blood–brain barrier compared to amphetamines, due to the presence of the beta-keto group, which increases their polarity. However, pyrrolidine derivatives are an exception, as their pyrrolidine ring lowers polarity, enhancing their ability to penetrate the blood–brain barrier. Understanding the metabolism of synthetic cathinones is crucial for proper identification in forensic toxicology cases.

The metabolic processes of synthetic cathinones are divided into two main phases:

- Phase I metabolism, which involves oxidation, reduction, and hydrolysis reactions, increases the molecule’s polarity by introducing functional groups.
- Phase II metabolism, which aims to make Phase I metabolites more water soluble for easier excretion. This process includes glucuronidation, sulfation, and reactions involving glutathione, N-acetylation, or methylation.

Cathinone and its synthetic derivatives undergo both Phase I and Phase II metabolism, primarily mediated by cytochrome P450 (CYP) enzyme in the first phase. However, many synthetic cathinones are also excreted unchanged in the urine [34,137,138]. The specific metabolic pathways vary according to the chemical structure of each cathinone, resulting in differences across groups of compounds, while being similar within each group.

### 5.1. Mephedrone Metabolism

Mephedrone, a N-alkyl synthetic cathinone, undergoes a series of Phase I reactions including

- N-dealkylation to form a primary amine,
- Reduction of the β-ketone group to produce an alcohol,
- Aromatic hydroxylation when halogen substituents are present on the aromatic ring, and
- Hydroxylation of the alkyl side chain (if present) or the α-alkyl side chain, which may lead to further oxidation into carboxylic acids.

**Figure 26:**
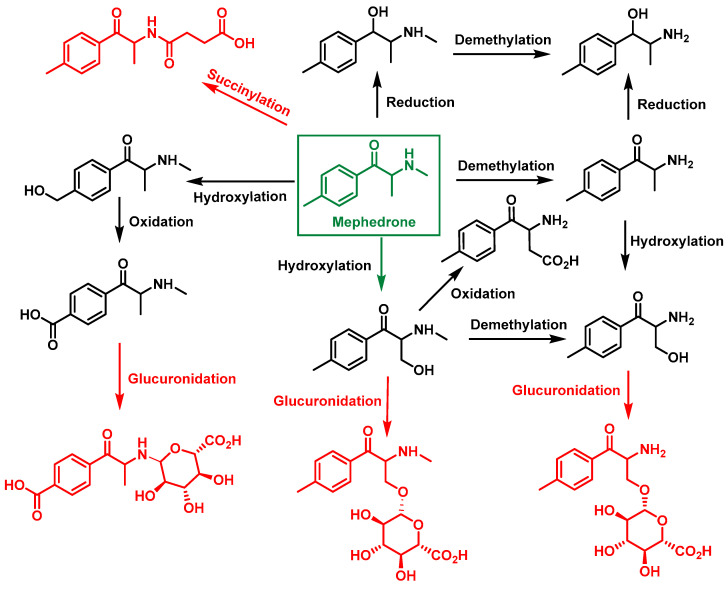
Proposed metabolic pathways for mephedrone metabolism in humans (showing Phase I in black and Phase II in red).

After Phase I, these metabolites can be subjected to Phase II reactions, typically glucuronidation or succinylation. Mephedrone’s metabolism has been studied both in vivo and in vitro, revealing its complex metabolic pathways [139,140,141] (Figure 26).

### 5.2. Methylone Metabolism

The metabolism of methylone, another synthetic cathinone, has been thoroughly studied using liquid chromatography–electrospray ionization mass spectrometry (LC–ESI/MS) and gas chromatography–mass spectrometry (GC–MS) [34,142,143]. Key reactions in Phase I metabolism include

- Demethylation followed by O-methylation at the 3,4-methylenedioxy ring,
- N-dealkylation to yield a primary amine, and
- Reduction of the β-ketone group to form the corresponding alcohol.

**Figure 27:**
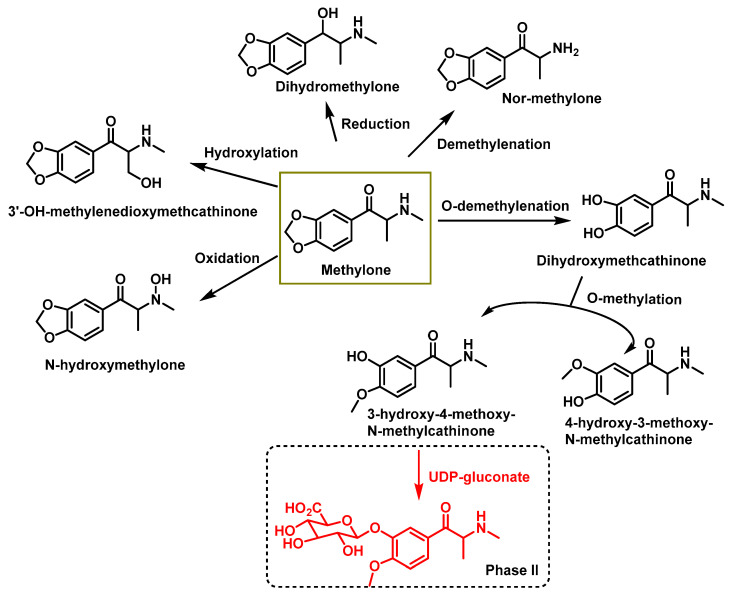
Metabolites identified in the literature for methylone using cell cultures and animal models.

These metabolites can then undergo Phase II processes, such as glucuronidation and sulfation. Studies on both human and rat urine have confirmed these pathways [142,144] (Figure 27).

### 5.3. Methylenedioxypyrovalerone (MDPV) Metabolism

The metabolism of MDPV, which contains a pyrrolidine group, has been investigated through in vitro studies. Strano et al., 2010, identified MDPV metabolites using GC–MS/EI and LC–QTOF, analyzing derivatives formed during metabolic reactions [145]. The major Phase I metabolites include catecholpyrovalerone and methylcatecholpyrovalerone, produced by the opening of the methylenedioxy ring followed by demethylation to form catechol, and subsequent methylation by the enzyme catechol-O-methyltransferase [15,146].

**Figure 28:**
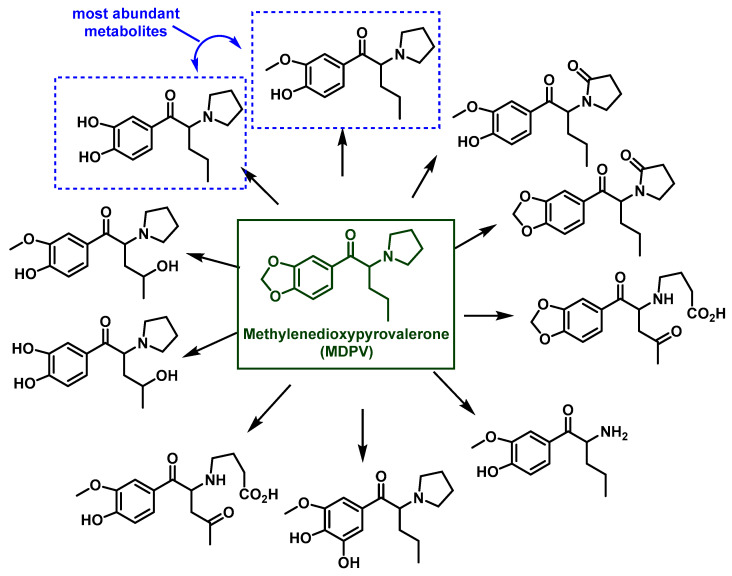
Chemical structures of MDPV metabolites observed in cell cultures and animal studies (highlighting the most abundant metabolites in blue).

In Phase II metabolism, MDPV metabolites undergo conjugation with glucuronic acid and sulfate, with approximately 40% and 50% of metabolites being excreted in these forms, respectively [147] (Figure 28).

## 6. Analytical Detection of Synthetic Cathinones in Biological Samples

Once ingested, synthetic cathinones can be detected in various biological matrices, providing valuable information for medical professionals and law enforcement, as well as insights into possible metabolites. Common matrices used for detection include urine [148], blood, hair [105,149,150], and saliva [151,152,153], while less frequently used matrices include vitreous humor [154] or meconium [155]. Table 3 outlines the advantages and disadvantages of each biological matrix for cathinone detection.

The choice of matrix depends on the intended purpose of the analysis. For identifying recent drug consumption (within the last 6–48 h), blood or saliva are the most appropriate matrices. For detecting drug use over the past few days, urine is preferred, while hair samples are ideal for long-term detection, allowing retrospective analysis over several months. However, a negative result in one matrix does not confirm the absence of drug use, as it only indicates the substance was not present in that particular sample at the time of testing. The most effective approach to monitor cathinone consumption is simultaneous analysis of multiple biological samples.

Given the rising number of synthetic cathinones detected in various biological matrices, it is critical to develop reliable and versatile tools that can quickly identify these substances. After consumption, cathinones are present in the body in both their metabolized and unmetabolized forms, with concentrations varying based on the dose, time since ingestion, and the body’s metabolism. Among the various biological matrices, urine remains the most widely used for detecting synthetic cathinones [156,157,158]. Several techniques are used for cathinone detection:

- Gas Chromatography–Mass Spectrometry (GC–MS): This method requires cathinone derivatization before analysis.
- Liquid Chromatography–Mass Spectrometry (LC–MS/MS) and Liquid Chromatography–High-Resolution Mass Spectrometry (LC–HRMS): These are commonly employed for high-efficiency detection.
- Electrochemical techniques: Emerging methods for cathinone detection.

Among these, HPLC and GC–MS are the most widely used techniques for both qualitative and quantitative analysis of synthetic cathinones, as well as for detecting adulterants commonly present in illicit drug formulations.

**Figure 29:**
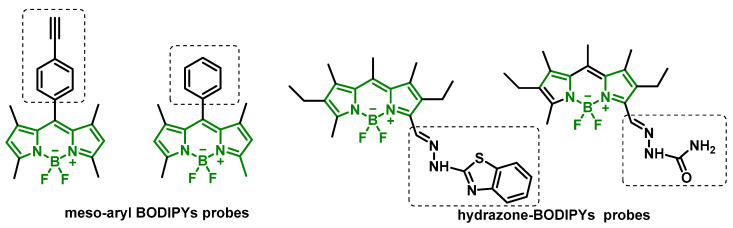
Meso-aryl BODIPYs and Hydrazone-BODIPY Probes for Cathinone Detection.

Innovative detection strategies are also being explored. Hernandez-Contreras et al., 2022, proposed using meso-arylic BODIPY probes combined with Cu(II) to detect cathinones in water and oral fluid, based on color changes and fluorescence [159]. The proposed mechanism involves cathinone-induced reduction of Cu(II) to Cu(I), triggering structural changes in BODIPY. Similarly, Rodríguez-Nuévalos et al., 2022, developed colorimetric and fluorescent hydrazone-BODIPY probes for cathinone detection in the presence of Cu(II), based on fluorescence quenching [160] (Figure 29).

The regulation of synthetic cathinones remains challenging due to the rapid emergence of new analogs. Minor modifications to the chemical structure allow new variants to bypass existing legal controls, potentially leading to more harmful substances entering the illicit drug market [5].

## 7. Toxicity of Synthetic Cathinones

Synthetic cathinones are known to produce a range of psychoactive effects, including intense euphoria, increased energy and concentration, talkativeness, empathy, and heightened sexual drive [161]. However, these desirable effects are often accompanied by adverse reactions, which vary among users. Some individuals may experience prolonged panic attacks, tremors, agitation, insomnia, nausea, headaches, tinnitus, vertigo, muscle spasms, dizziness, increased heart rate, altered vision, confusion, short-term memory impairment, anhedonia, depression, suicidal thoughts, psychosis, and even the development of tolerance and dependence [162].

**Figure 30:**
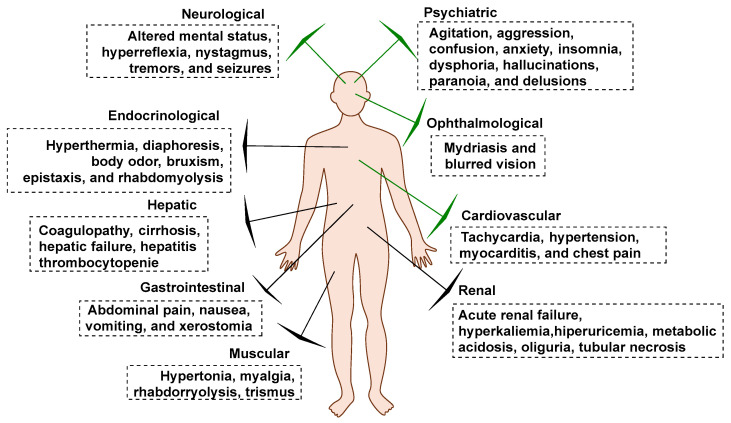
Major clinical manifestations related to synthetic cathinone intoxication.

From a clinical perspective, synthetic cathinone intoxication can result in a wide array of negative effects, with neurological, psychiatric, and cardiovascular symptoms being the most prominent [5,24,163]. These major clinical manifestations of cathinone poisoning are illustrated in Figure 30.

In addition to the common neurological and psychiatric symptoms, gastrointestinal, hepatic, hematological, musculoskeletal, pulmonary, and renal complications can also arise [164,165,166,167,168,169,170,171]. Some of these adverse effects can be part of broader clinical syndromes, including sympathomimetic toxidrome, hallucinatory toxidrome, and excited/agitated delirium syndrome.

The abuse of synthetic cathinones can lead to multiorgan failure and even death, which may result from:

- The toxicity of one or more substances consumed,
- Suicide, possibly triggered by severe psychiatric effects, or
- Accidents caused by driving under the influence of these substances [63].

## 8. Antimicrobial Properties of *Catha edulis* Extracts

The naturally occurring cathinone found in *Catha edulis* (khat) has been widely studied for its stimulant effects [2]. However, new research suggests that cathinone and other alkaloids from the plant may also have antimicrobial activity [172]. The antimicrobial activity of *Catha edulis* against MDR pathogens is of particular importance given the global increase in antibiotic resistance [173]. MDR bacteria pose a major public health challenge and natural products such as khat offer a promising source of new antimicrobial agents. Cathinone and its related compounds may inhibit bacterial growth by different mechanisms than conventional antibiotics, making them valuable candidates for further research in the development of new antimicrobial agents [173].

Studies investigating the antimicrobial properties of khat extracts have shown activity against multidrug-resistant (MDR) bacteria such as *S. aureus* and *E. coli*. The results appear to vary depending on the solvents used in the extraction, suggesting that specific bioactive compounds in *C. edulis* are responsible for these effects [174]. While cathinone is the primary psychoactive compound, it remains unclear whether it or related alkaloids are directly responsible for the observed antimicrobial effects. Other phytochemicals in *C. edulis*, such as flavonoids or glycosides, could contribute to this effect [4,175]. Different solvents can extract different concentrations of active compounds, resulting in different levels of antimicrobial activity [176]. In a disk diffusion test, the methanolic crude extract of *C. edulis* (0.1 mg protein/disk) showed antibacterial activity against *Brevundimonas diminuta* (19 ± 2.3 mm), *Micrococcus luteus* (22 ± 3.1 mm) and *Bacillus megaterium* (16 ± 0.7 mm). However, it showed no significant effect against a clinical isolate of *E. coli*, with an inhibition zone (<11 mm) below the EUCAST breakpoint of 14 mm, whereas gentamicin (positive control) produced a zone >18 mm [177]. Methanolic and DMSO extracts of *C. edulis* (100 μg) showed significant antimicrobial activity, with zones of inhibition exceeding EUCAST breakpoints against several bacterial isolates, including antibiotic-resistant strains of *S. aureus*, *E. coli* and *Pseudomonas aeruginosa*. In particular, the methanolic extract showed an inhibition zone of 29 mm against *Streptococcus pyogenes*, a strain resistant to most antibiotics tested [174]. Using microbroth dilution and agar-well diffusion methods and determination of the minimum inhibitory concentration (MIC) and minimum bactericidal concentration (MBC), the aqueous, acetone and methanol extracts of *Catha edulis* showed no activity against *E. coli*, *Pseudomonas aeruginosa* or *Candida albicans* and limited activity against *Bacillus cereus* and *S. aureus*, compared to standard antimicrobials. The lack of activity against *P. aeruginosa* could be due to the fact that the extracts are not able to disrupt the cytoplasmic membrane permeability of the pathogen [176]. This suggests that future research should focus on optimizing extraction methods to isolate and identify the compounds with the strongest antimicrobial properties.

Beyond its antimicrobial properties, cathinone from *C. edulis* has been shown to inhibit viability and proliferation in various cell types in vitro, inducing ultrastructural changes consistent with apoptosis. These changes appear to be mediated by the mitochondrial apoptosis pathway, with an observed increase in pro-apoptotic Bax and decrease in anti-apoptotic Bcl-2 protein expression, along with elevated reactive oxygen species (ROS) production and activation of caspase cascades [178,179]. These findings suggest that cathinone may influence cell health beyond microbial inhibition, warranting further comparative phytochemical and clinical studies to better understand its effects on eukaryotic cells.

## 9. Nanotechnological Applications of *Catha edulis* Extracts

Recent research has explored the potential of *Catha edulis* (khat) leaf extracts in synthesizing nanoparticles with notable antimicrobial properties, particularly against antibiotic-resistant pathogens. One study optimized the bio-fabrication of silver nanoparticles (AgNPs) using *Catha edulis* extracts [180]. These AgNPs, which averaged 27–32 nm in size, showed significant antibacterial activity against methicillin-resistant *Staphylococcus aureus* (MRSA) and extended-spectrum beta-lactamase-producing *Escherichia coli*. This enhanced efficacy is attributed to the AgNPs’ high surface area, which facilitates interaction with bacterial cell walls, resulting in microbial cell death. Interestingly, the silver ions alone displayed lower efficacy compared to the complete nanoparticle structure, highlighting the synergistic role of plant compounds in stabilizing and enhancing nanoparticle potency [180].

Other studies extended these findings to nanoparticles of copper oxide (CuO) and zinc oxide (ZnO), also synthesized using *Catha edulis* extracts [181,182]. These CuO and ZnO nanoparticles demonstrated similar antimicrobial benefits against a range of bacterial strains. The CuO nanoparticles, for instance, achieved bactericidal effects against multiple Gram-positive and Gram-negative bacteria, including resistant strains, and the plant’s bioactive compounds played a role in reducing and capping the nanoparticles, enhancing stability and bioactivity [182].

Recent studies have highlighted the nanotechnology applications of *Catha edulis* (khat) extracts for biomedical and environmental purposes. Song et al., 2024, investigated *Catha edulis* extract-loaded nanofibrous scaffolds in a diabetic wound healing model, where the extract was incorporated into polycaprolactone/gelatin scaffolds and then seeded with bone marrow-derived stem cells [183]. These scaffolds demonstrated significant wound healing efficacy in rats, promoting faster wound closure, increased vascular endothelial growth factor (VEGF) expression, and reduced oxidative stress markers, positioning them as a promising therapeutic option for diabetic wounds [183].

In another study, Lyu and Chen (2023) developed *Catha edulis* extract-loaded nanofibrous neural channels to aid sciatic nerve repair. These channels not only supported cellular survival and metabolic activity but also upregulated the genes for brain-derived neurotrophic factor (BDNF) and nerve growth factor (NGF), which are crucial for nerve regeneration [184].

Additionally, green synthesis methods have used *Catha edulis* leaf extracts to create magnetite and copper oxide nanoparticles for environmental applications, such as hexavalent chromium removal from water. These nanoparticles, synthesized through eco-friendly processes, exhibit high adsorption capacities, providing an efficient and sustainable method for treating water pollution [185,186].

## 10. Interactions Between Cathinone and Antibiotics

There is an intriguing overlap between the neuroactive properties of cathinone and research on antibiotics [187]. The dual nature of *Catha edulis* as a psychoactive and antimicrobial agent presents a unique therapeutic challenge. Studies have shown that β-lactam antibiotics, such as clavulanic acid, can modulate glutamatergic neurotransmission, which is also affected by cathinone [188]. To minimize unwanted neurological side effects while preserving antimicrobial activity, it may be necessary to isolate certain compounds from khat or modify them structurally. This approach could allow researchers to preserve the beneficial properties of these compounds without triggering the psychoactive effects commonly associated with cathinone. Exploring this potential synergy could have therapeutic implications for diseases associated with neurotransmitter dysregulation. However, it also poses potential risks when cathinone is co-administered with antibiotics, particularly if cathinone exacerbates side effects or leads to unpredictable pharmacodynamic outcomes when used with antibiotics in the clinical setting, which warrants further investigation [189,190].

## 11. Conclusions

The increasing use and abuse of synthetic cathinones continues to pose a significant public health problem due to their potent stimulant and hallucinogenic effects, which have led to them replacing more expensive illicit drugs such as MDMA, cocaine and amphetamines. These compounds are associated with serious cardiovascular, neurological and psychiatric effects, including addiction and death. Despite international efforts to control some of these substances, the constant emergence of new cathinone derivatives continues to pose new challenges for law enforcement, healthcare providers and forensic toxicologists.

This review highlights the complexity of regulating synthetic cathinones, particularly given their structural diversity and the existence of different enantiomers with different biological and toxicological profiles. The rapid turnover of new derivatives on the illicit market emphasizes the need for continuous monitoring and development of analytical methods to detect these substances in biological samples.

Apart from their psychoactive and toxicological effects, recent studies have shown that compounds derived from *Catha edulis* possess antimicrobial properties. This opens a new avenue for exploring their potential use in combating antimicrobial resistance (AMR), especially against MDR bacteria such as *S. aureus* and *E. coli*. The versatility of *Catha edulis* in nanotechnology have showed potential for both medical applications, like wound healing and nerve repair, and environmental remediation. Future research should aim to isolate and modify specific cathinone-derived compounds to exploit their antimicrobial properties while minimizing their psychoactive and toxicological risks.

## Figures and Tables

**Table 1:** Monoamine transporter inhibition.

	NET	DAT	SERT	DAT/SERT Ratio
	IC_50_ (µM) (95% CI)	IC_50_ (µM) (95% CI)	IC_50_ (µM) (95%CI)	
Cocaine (a)	0.451 (0.38–0.59)	0.768 (0.6–1.0)	2.37 (2.0–2.9)	3.1
MDMA (a),(c)	0.447 (0.33–0.60)	17 (12–24)	1.36 (1.0–2.0)	0.08
Methamphetamine (a),(c)	0.064 (0.04–0.09)	1.05 (0.74–1.5)	>10	>10
**NET > SERT > DAT selectivity**				
2,3-Dimethylmethcathnone (d)	0.53 (0.36–0.78)	7.4 (5.4–10.1)	1.2 (1.0–1.4)	0.16
2,4-Dimethylmethcathnone (d)	1.5 (1.1–2.0)	83 (65–105)	1.5 (1.0–2.2)	0.02
3,4-Dimethylmethcathnone (d)	0.45 (0.33–0.60)	9.4 (7.6–11.7)	1.1 (0.9–1.4)	0.12
4-Ethylmethcathinone (c)	2.5 (1.7–3.7)	31 (13–72)	4.3 (3.2–5.9)	0.14
Methedrone (b)	2.24 (1.4–3.5)	35 (15–79)	4.73 (3.2–6.9)	0.14
4-Methylmethcathinone (d)	0.26 (0.19–0.35)	5.7 (4.5–7.2)	3.6 (2.8–4.6)	0.63
4-Bromomethcathinone (c)	0.41 (0.30–0.57)	5.6 (2.7–12)	2.2 (1.7–2.8)	0.4
**NET > DAT ≥ SERT selectivity**				
Buthylone (a)	2.02 (1.5–2.7)	2.90 (2.5–3.4)	6.22 (4.3–9.0)	2.1
Ethylone (a)	2.54 (2.0–3.2)	5.68 (4.9–6.5)	4.46 (3.8–5.2)	0.8
Mephedrone (a),(c)	0.254 (0.22–0.30)	3.31 (2.6–4.2)	4.64 (3.7–5.9)	1.4
4-Methylethcathinone (b)	2.23 (1.6–3.2)	4.28 (3.4–5.4)	7.93 (3.5–18)	1.85
3-Methylmethcathinone (d)	0.27 (0.21–0.36)	2.6 (2.0–3.3)	9.5 (6.9–13.2)	3.7
Methylone (a)	0.542(0.39–0.75)	4.82 (3.8–6.1)	15.5 (10–26)	3.3
Naphyrone (a),(c)	0.25 (0.20–0.32)	0.47 (0.40–0.55)	0.96 (0.85–1.09)	2.0
Pentylone (b)	0.99 (0.72–1.4)	1.34 (1.0–1.7)	8.37 (5.4–13)	6.2
**NET > DAT ≫ SERT selectivity**				
Buphedrone (b)	0.65 (0.51–0.81)	4.24 (3.3–5.5)	70 (2–2700)	>10
Cathinone (a)	0.199 (0.15–0.26)	14.0 (10–20)	>100	>10
N,N-Dimethylcathinone (b)	7.71 (5–12)	27 (21–36)	>500	>10
Ethcathinone (b)	0.44 (0.34–0.56)	5.00 (3.7–6.8)	48 (4–529)	9.6
Flephedrone (a),(c)	0.246 (0.16–0.37)	6.35 (4.2–9.5)	>10	5.8
3-Fluoromethcathinone (b)	0.19 (0.13–0.29)	1.7 (1.0–3.0)	56 (7–472)	>10
Methcathinone (a),(c)	0.085 (0.06–0.17)	1.12 (0.83–1.5)	>10	>10
Pentedrone (b)	0.61 (0.52–0.72)	2.50 (2.0–3.2)	135 (5–3700)	>10
**Highly potent NET and DAT blockers**				
MDPBP (c)	16 (0.11–0.24)	0.11 (0.07–0.16)	15 (5.4–39)	132
MDPPP (c)	0.97 (0.62–1.5)	0.53 (0.27–1.1)	75 (49–114)	141
MDPV (a),(c)	0.044 (0.03–0.07)	0.031 (0.03–0.04)	9.30 (6.8–12.8)	>100
Pyrovalerone (a),(c)	0.043 (0.03–0.06)	0.035 (0.03–0.04)	13.0 (10.8–15.8)	>100
α-PVP (c)	0.02 (0.01–0.03)	0.04 (0.01–0.1)	>100	>1000

The values are the means of three to four independent experiments and 95% confidence intervals (CIs). (a) [19]; (b) [130]; (c) [61]; (d) [136].

**Table 2:** Monoamine transporter and receptor binding affinities.

	5-HT_1A_	5-HT_2A_	5-HT_2C_	α_1A_	α_2A_
Cocaine (a)	>20	>13	>13	>6	>20
MDMA (a),(c)	12.2 ± 0.8	7.8 ± 2.4	>13	>6	15.0 ± 10
Methamphetamine (a),(c)	8.07 ± 0.75	>13	>13	>6	6.1 ± 1.6
**NET > SERT > DAT selectivity**					
2,3-Dimethylmethcathnone (d)	>17	0.64 ± 0.19	2.4 ± 0.9	0.78 ± 0.10	3.0 ± 0.1
2,4-Dimethylmethcathnone (d)	15 ± 3	1.3 ± 0.1	1.3 ± 0.3	0.16 ± 0.02	3.0 ± 0.3
3,4-Dimethylmethcathnone (d)	>17	1.9 ± 0.3	1.5 ± 0.2	1.9 ± 0.3	3.5 ± 0.2
4-Ethylmethcathinone (c)	>20	6.5 ± 0.9	9.6 ± 0.4	8.4 ± 3.4	21.1 ± 7.6
Methedrone (b)	>20	>13	>13	>6	>25
4-Methylmethcathinone (d)	>17	1.6 ± 0.2	8.1 ± 5.4	1.1 ± 0.1	11 ± 1
4-Bromomethcathinone (c)	>20	3.2 ± 0.6	>13	8.2 ± 3.0	12.7 ± 0.2
**NET > DAT ≥ SERT selectivity**					
Buthylone (a)	>20	>13	>13	>6	>25
Ethylone (a)	17.0 ± 2.4	>13	>13	>6	>25
Mephedrone (a),(c)	>20	2.1 ± 0.7	>13	3.48 ± 2.2	11.0 ± 5.0
4-Methylethcathinone (b)	>20	3.8 ± 1.6	5.2 ± 0,3	>6	>18
3-Methylmethcathinone (d)	4.8 ± 0.5	3.4 ± 0.8	3.6 ± 1.0	7.9 ± 0.2	1.1 ± 0.1
Methylone (a)	>20	>13	>13	>6	>20
Naphyrone (a),(c)	6.00 ± 0.21	11 ± 2.2	>13	>6	8.0 ± 2.8
Pentylone (b)	>18	>13	>13	>6	>25
**NET > DAT ≫ SERTselectivity**					
Buphedrone (b)	>18	>13	>13	>6	23.9 ± 4.2
Cathinone (a)	>20	>13	>13	5.40 ± 1.1	8.9 ± 2.7
N,N-Dimethylcathinone (b)	>18	6.5 ± 0.8	6.5 ± 0.8	>6	25.4 ± 11
Ethcathinone (b)	8.5 ± 1.1	>13	9.3 ± 0.2	>6	15.5 ± 1.9
Flephedrone (a),(c)	>20	1.4 ± 0.6	>13	1.52 ± 0.05	>20
3-Fluoromethcathinone (b)	>18	>13	6.1 ± 2.2	>6	10 ± 2.2
Methcathinone (a),(c)	2.7 ± 3.5	3.0 ± 0.6	>13	3.93 ± 1.3	11.9 ± 3.9
Pentedrone (b)	>18	>13	>13	>6	35.4 ± 16
**Highly potent NET and DAT blockers**					
MDPBP (c)	13.0 ± 0.02	>13	>13	>4.9	9.4 ± 1.6
MDPPP (c)	2.5 ± 0.3	7.5 ± 0.1	>13	>15	13.9 ± 0.9
MDPV (a),(c)	10.29 ± 4.7	>13	>13	>6	>20
Pyrovalerone (a),(c)	13.4 ± 2.1	>13	>13	>6	>20
α-PVP (c)	5.2 ± 0.1	>13	>13	>15	>20

The values are the means of three to four independent experiments and 95% confidence intervals (CIs). (a) [19]; (b) [130]; (c) [61]; (d) [137].

**Table 3:** Advantages and disadvantages of biological matrices for synthetic cathinone detection.

Biological Matrix	Advantages	Disadvantages
Urine	Non-invasive sampling High volume High concentrations of analytes and metabolites	Easily manipulated Unstable if not preserved in some suitable conditions
Blood	There is a correlation between the concentration found and the amount of drug consumed	Invasive extraction method Specialized personnel required Limited volume Unstable if not preserved in some suitable conditions
Saliva	Difficult handling Non-invasive sampling Whole drug (not metabolized)	Low volume Sample contamination food waste
Hair	Simple sampling Difficult to manipulate Non-invasive sampling Allows drug detection consumed and their metabolites	Complex sample treatment Possible contamination of the sample for use of products cosmetics.
